# The Epidemiology of COVID-19 Vaccine-Induced Myocarditis

**DOI:** 10.1155/2024/4470326

**Published:** 2024-04-18

**Authors:** Christos Costa, Foteini Moniati

**Affiliations:** ^1^Imperial College London, London, UK; ^2^Queen Mary University of London, Barts and the London School of Medicine and Dentistry, London, UK

## Abstract

**Background:**

In December 2019, the emergence of severe acute respiratory syndrome coronavirus-2 (SARS-CoV-2) led to the COVID-19 pandemic, with millions of deaths worldwide. Vaccine breakthroughs in late 2020 resulted in the authorization of COVID-19 vaccines. While these vaccines have demonstrated efficacy, evidence from vaccine safety monitoring systems around the globe supported a causal association between COVID-19 vaccines, in particular those using mRNA technology, i.e., Moderna's mRNA-1273 and Pfizer-BioNTech's BNT162b2, and myocarditis.

**Objective:**

This paper aims to investigate the epidemiology of mRNA COVID-19 vaccine-induced myocarditis, including age, ethnicity, and gender associations with these vaccines. It also discusses the immunopathophysiological mechanisms of mRNA COVID-19 vaccine-associated myocarditis and outlines principles of diagnosis, clinical presentation, and management.

**Methods:**

A literature review was conducted using PubMed, Embase, and Queen Mary University of London Library Services databases. Search terms included “myocarditis,” “coronavirus disease 2019,” “SARS-CoV-2,” “mRNA Covid-19 vaccines,” “Covid vaccine-associated myocarditis,” “epidemiology,” “potential mechanisms,” “myocarditis diagnosis,” and “myocarditis management.”

**Results:**

While the definite mechanism of mRNA COVID-19 vaccine-associated myocarditis remains ambiguous, potential mechanisms include molecular mimicry of spike proteins and activation of the adaptive immune response with dysregulated cytokine expression. Male predominance in COVID-19 vaccine-induced myocarditis may be attributed to sex hormones, variations in inflammatory reactions, coagulation states based on gender, and female-specific protective factors. Moreover, an analysis of diagnostic and management strategies reveals a lack of consensus on acute patient presentation management.

**Conclusion:**

In contrast to viral infections that stand as the predominant etiological factor for myocarditis with more severe consequences, the mRNA COVID-19 vaccination elicits a mild and self-limiting manifestation of the condition. There is currently insufficient evidence to confirm the definite underlying mechanism of COVID-19 vaccine-associated myocarditis. Further research is needed to develop preventive and therapeutic solutions in this context.

## 1. Introduction

### 1.1. Classic Myocarditis

#### 1.1.1. Classification and Aetiology

**Figure 1:**
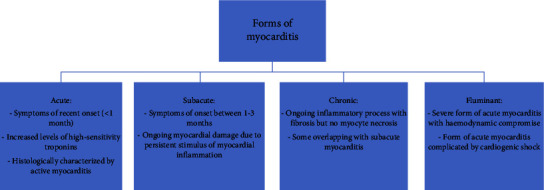
Summary of the different forms of myocarditis classified as acute, subacute, chronic, and fulminant.

Myocarditis signifies inflammation within the myocardium, the heart's middle layer, leading to degeneration and eventual necrosis [1]. It can manifest as acute, subacute, chronic, or fulminant myocarditis. Acute myocarditis presents symptoms within a month, accompanied by elevated high-sensitivity troponin levels. According to the later-discussed Dallas criteria, it exhibits histological features of active myocarditis characterized by infiltrating mononucleated cells and monocyte necrosis [2]. Subacute myocarditis emerges when ongoing myocardial inflammation leads to sustained myocardial damage, with symptoms arising between one and three months. Chronic myocarditis denotes a persistent inflammatory process featuring fibrosis but no myocyte necrosis, often overlapping with subacute myocarditis [3]. Fulminant myocarditis, associated with severe acute myocarditis and hemodynamic compromise, frequently leads to cardiogenic shock. Endomyocardial biopsy (EMB) reveals diffuse inflammatory infiltrates, as elaborated later [2]. As far as the aetiology of myocarditis is concerned, the causes can be classified into both infectious and noninfectious categories [2, 4].  Figure 1 summarizes the myocarditis forms mentioned [3].

#### 1.1.2. Epidemiology

The true incidence of myocarditis is challenging to quantify due to the variability in clinical presentation [5]. While endomyocardial biopsy (EMB) is the gold standard for definitive diagnosis, its invasive nature limits its use [6]. Electroanatomic mapping (EAM) has emerged as an alternative for diagnosing myocarditis, offering improved sensitivity and reduced false-negative rates compared with EMB [7].

Before the emergence of COVID-19, the Global Burden of Cardiovascular Disease reported an annual prevalence of 4.4 cases per 100,000 people aged 35–39 years in women and 6.1 cases in men, with corresponding mortality rates of 0.1 and 0.2 per 100,000 people, respectively [8]. However, during the first 8 months of the pandemic, the prevalence of excess cardiovascular deaths in England and Wales rose to 12 per 100,000 people, while, in England, there was a marked 8% increase in acute cardiovascular disease deaths [9]. Concurrently, in the United States, hypertensive and ischemic heart disease rose more rapidly in the first 10 months of the COVID-19 pandemic compared with the year before [10].

A study published in February 2020, examining sex differences in myocarditis clinical presentation, found that the majority of patients with myocarditis were male (82%) and young adults (average age: men: 40 ± 16; women: 40 ± 17) [11].

#### 1.1.3. Pathophysiology

The academic community extensively investigates the molecular and cellular pathophysiology of postviral myocarditis in animal models [12]. A simplified three-stage process elucidates cellular and molecular pathogenesis.

1. Immune Activation: in the initial stage, pathogens, typically viruses or toxins, injure cardiac myocytes, exposing intracellular antigens such as cardiac myosin and activating the innate immune system [13, 14]. This stage involves upregulating Toll-like receptor 4 (TLR4) on macrophages, maturing antigen-presenting cells (APCs), and releasing pro-inflammatory cytokines, including interleukin-1 (IL-1) [15].
2. Inflammatory Response: in the second stage, CD4+ T-lymphocytes play a key role, producing cytokines that lead to a Th1/Th2/Th17/Th22 (T-helper)-biased immune response [16]. B-lymphocytes also contribute to inflammation by producing antibodies.
3. Outcome Variation: in the third stage, most patients experience a reduced immune response, leading to viral clearance facilitated by cytotoxic CD8+ T-lymphocytes [17]. However, in some cases, viral clearance remains elusive, resulting in persistent myocyte injury [17].

The pathways implicated in the pathogenesis of vaccine-associated myocarditis closely resemble those observed in viral myocarditis discussed above, suggesting a shared pathophysiology. In this case, however, tumour necrosis factor-alpha (TNF-*α*), interferon gamma (IFN-*γ*), IL-6, and IL-1 are the inflammatory cytokines involved, with genetic predispositions to IL-6-induced inflammation thought to exacerbate vaccine reactions [18]. Furthermore, in contrast to classic myocarditis, the spike protein utilized in the vaccine could induce molecular mimicry with *α*-myosin, akin to phenomena observed in certain COVID-19 infections [19]. Additionally, there is a plausible autoimmune aspect through molecular mimicry [18].

#### 1.1.4. Evaluation and Diagnosis

**Figure 2:**
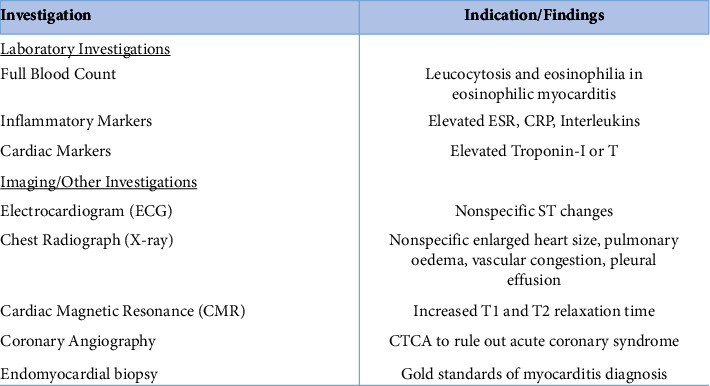
Overview of the diagnostic investigations of myocarditis and respective findings. ESR: erythrocyte sedimentation rate; CRP: C-reactive protein.

Due to the overlap in symptoms with other clinical presentations, the diagnosis of myocarditis is often challenging. It is, therefore, important to highlight that a preceding acute febrile illness, symptoms of connective tissue disease, and a viral infection should always lead to suspicion [20–22].  Figure 2 provides an overview of the investigations used in the diagnosis of myocarditis and respective findings [21, 23].

#### 1.1.5. Treatment

Determining the aetiology in classic myocarditis cases using a multidisciplinary approach is crucial for effective management. For example, myocarditis caused by immune-mediated diseases requires immunosuppressants (e.g., corticosteroids), and viral-induced myocarditis necessitates anti-infective agents [24]. However, when the cause is unknown, treatment primarily focuses on supportive care, including managing complications such as heart failure or arrhythmias [25].

### 1.2. COVID-19 Pandemic and Vaccine Development

#### 1.2.1. The Coronavirus Disease 2019 (COVID-19)

As of January 2021, the COVID-19 pandemic had a significant global impact, with over 100 million infections and 2 million deaths, affecting the economy, psychology, and health [1, 26]. The FDA's emergency use authorization in December 2020 for two mRNA vaccines, Pfizer-BioNTech and Moderna, was pivotal. These vaccines demonstrated high safety and effectiveness, with reported rates of 94-95% efficacy after two doses [27]. However, concerns about potential side effects due to the rapid vaccine development increased hesitancy toward mRNA vaccine acceptance [26, 28].

#### 1.2.2. The mRNA Vaccine Platform

**Figure 3:**
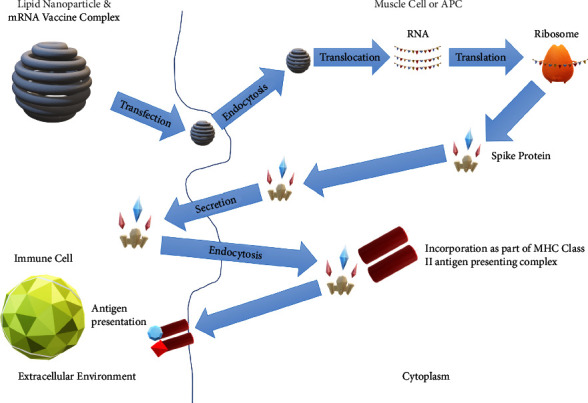
Visual representation of the mechanism of action of the mRNA vaccine: the lipid nanoparticle and mRNA vaccine complex enter the muscle cell or APC by endocytosis. Translocation and translation to spike proteins take place in the ribosomes. Secretion of the spike protein into the extracellular environment, internalization into APCs via endocytosis, and incorporation as part of MHC class II take place. Antibody and cell-mediated immunity against SARS-CoV-2 take place.

The discussed vaccines are lipid nanoparticle-encapsulated mRNA vaccines, encoding prefusion stabilized spike proteins [28, 29]. Nanoparticles are extensively studied in vaccine development and serve as common vectors for in vivo RNA delivery, preventing mRNA degradation and facilitating endocytosis [30]. Positively charged lipid nanoparticles aid mRNA delivery to the negatively charged cell membrane, enabling cytoplasmic endocytosis. Once inside, mRNA is released, leading to spike protein translation in ribosomes. The final steps involve spike protein secretion, internalization by APCs, and incorporation into the major histocompatibility complex (MHC) class II antigen-presenting complex [31]. This process generates an adaptive immune response, fostering antibody and cell-mediated immunity against SARS-CoV-2 spike proteins [32, 33].  Figure 3 illustrates the mRNA vaccine mechanism [34].

### 1.3. COVID-19 Vaccine-Induced Myocarditis

**Figure 4:**
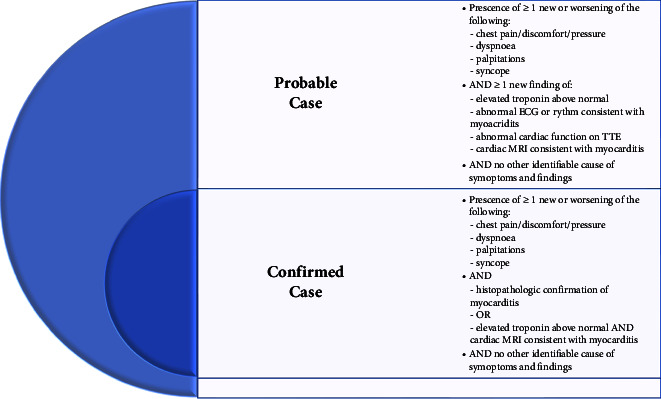
Illustration of the CDC definition of myocarditis. ECG: electrocardiogram; TTE: transthoracic echocardiogram; MRI: magnetic resonance imaging.

The extensive COVID-19 vaccination program, launched in December 2020, prompted the FDA and Centers for Disease Control and Prevention (CDC) to assess vaccine side effects through the Vaccine Adverse Event Reporting System (VAERS), which encourages voluntary reporting of postvaccine side effects [4, 35]. The CDC was the first to link the two mRNA COVID-19 vaccines with myocarditis, estimating an incidence of 0.48 per 100,000 in the general population and 1.2 per 100,000 in individuals aged 18 to 29 [35]. In April 2021, the CDC classified patients' adverse vaccine reactions according to working case definitions for probable or confirmed myocarditis.  Figure 4 illustrates the classification [36].

As of June 2021, in a cohort of 300 million mRNA-vaccinated patients, there were 1226 reports of probable myocarditis [37]. Notably, 79% of these cases occurred in males, with a median age of 24 years, and symptoms typically emerged about a week after the second vaccine dose. Common symptoms included chest pain and dyspnoea, with or without palpitations. Among male adolescents, 86% experienced chest pain, 64% had elevated cardiac enzymes, and 61% exhibited ECG changes [37]. Among 323 confirmed myocarditis cases, 310 patients were hospitalized and later discharged with symptom resolution [37]. However, the reasons for this male predominance remain unknown, despite recognition by the CDC.

Although the exact mechanisms behind mRNA COVID-19 vaccine-related myocarditis are unclear, several hypotheses have been proposed [38]. Some researchers suggest direct viral invasion as a potential mechanism, while others propose host cell inflammatory responses. Notably, cardiac histopathology studies have reported a lack of diffuse lymphocytic myocarditis, which is characteristic of the classic presentation [39, 40]. Research on inflammatory infiltrates suggests that an exaggerated innate immune system response, increased pro-inflammatory cytokines, and endothelial dysfunction may contribute to the pathophysiology of COVID-19 vaccine-related myocarditis [18]. Certain individuals with genetic predispositions may experience hyperimmune responses to mRNA, leading to both aberrant innate and adaptive immune responses [18]. TLR-expressing cells exposed to RNA may express activation markers and cytokines, with potential differences when exposed to modified RNA compared with unmodified RNA [41]. This may trigger the immune system to detect mRNA in the vaccine as an antigen, leading to immune activation and pro-inflammatory cascades [18]. Additionally, stress, ischemia, and hypoxia-induced myocardial injury are suggested as alternative mechanisms [42].

Long-term outcomes, cardiac function, and implications in affected patients are still unknown, necessitating ongoing follow-up [43–45]. Currently, there is no consensus on the management of COVID-19 vaccine-induced myocarditis, raising questions about whether the traditional myocarditis treatment plan should be followed in these cases [46, 47].

This paper aims to review existing literature and secondary data on mRNA COVID-19 vaccine-induced myocarditis, exploring its epidemiology, including age, ethnicity, and gender associations, with a focus on male predominance. The paper will also analyse the immunopathophysiological mechanisms and outline the principles of diagnosis, clinical presentation, and management, emphasizing early identification and timely therapy.

## 2. Methodology

A literature review was undertaken to examine the association between mRNA vaccination against SARS-CoV-2 and myocarditis. The primary databases employed in the sourcing of material in this review were PubMed, Embase, and Queen Mary University of London Library Services. The articles were selected on the basis that they were peer-reviewed, reliable, and published between January 2000 and December 2023. The terms employed in the research include “myocarditis,” “coronavirus disease 2019,” “SARS-CoV-2,” “mRNA Covid-19 vaccines,” “Covid vaccine-associated myocarditis,” “epidemiology,” “potential mechanisms,” “myocarditis diagnosis,” and “myocarditis management.” The inclusion and exclusion criteria used in the database search are summarized in Table 1.

## 3. Results

During the literature search, we identified a total of 107 articles and evaluated them to determine their alignment with our inclusion and exclusion criteria. Among these, 8 of the 107 papers met our criteria and were subsequently subjected to a thorough review.

A search of electronic databases, including the World Health Organization's (WHO) Global Literature on Coronavirus Disease, identified 22 eligible randomized controlled trials and observational studies reporting the risk of COVID-19 vaccines and myocarditis on a global scale [48]. Between December 2019 and May 2022, among 58 million people, 55.5 million received the COVID-19 vaccination. Only 12 studies received Moderna, Pfizer-BioNTech, or mixed mRNA vaccines. Among 37.6 million mRNA vaccine recipients, 588 developed myocarditis, with 66% being men and number of deaths totalling 3. The median follow-up from vaccination to myocarditis was 28 days.  Table 2 shows the baseline study characteristics.

### 3.1. Risk of Myocarditis Associated with the COVID-19 Vaccination

Evaluating the risk of COVID-19 vaccine-induced myocarditis involves considering several factors. Firstly, there is the background risk of myocarditis in different geographic locations, and over time, it is influenced by the viral pandemic [43]. Current data primarily capture cases with more than minimal symptoms, leaving the full extent unknown [48].

The meta-analysis following the systematic review reported an increasing risk of myocarditis associated with younger age. Even though there was an increased association between the risk of myocarditis, mRNA vaccines, and male gender in the USA, the association was not statistically significant. In addition, the risk of hospitalization and death following COVID-19 vaccine-induced myocarditis was low, and it did not result in more serious outcomes compared with those unrelated to vaccination.

### 3.2. Age-Specific Risk of Myocarditis Associated with the COVID-19 Vaccination

The prevalence of COVID-19 vaccine-induced myocarditis risk, especially in young males, remains uncertain [46, 47]. Nevertheless, assessing population-based risk estimates for vaccine-related myocarditis across age, gender, and ethnicity is crucial, especially as vaccination efforts target young individuals and additional doses are administered.

Regarding age, similar results have also been reported by VAERS in the USA.  Table 3 illustrates the number of observed myocarditis cases based on gender and age in a 7-day risk window following the second dose of mRNA vaccination through June 11, 2021 [60]. The observed cases of myocarditis demonstrate a higher incidence in males than females and higher at younger ages than older ones with the highlight being 18–24 years of age [60].

The crude reported cases of myocarditis including death reports per million mRNA COVID-19 vaccine doses are presented in Table 4. According to the results published by VAERS in the USA through June 11, 2021, male rates per million doses are higher than female rates [60].

Pharmacovigilance systems in France and other countries, including Israel, reported similar results [61–63]. Population-based cohort studies in European countries such as Denmark and the United Kingdom also showed a higher risk associated with the Moderna vaccine [56, 64].

This increased risk, particularly notable among individuals aged 12 to 39 in Denmark [65], remained low overall, even among young recipients. These findings highlight an elevated risk of COVID-19 vaccine-related myocarditis occurring within one week after mRNA vaccination, especially following the second dose of the Moderna vaccine [56].

Current studies suggest that while the risk of myocarditis is elevated when the mRNA vaccine is given as a booster dose, this is still lower than after the second dose [66, 67]. The notable difference in risk was evident, especially after the mRNA-1273 vaccine, when comparing the second dose to the booster dose. According to Milano et al., the mRNA content in mRNA-1273 during booster administration is 50 micrograms, which is half the dosage administered during the priming phase [68]. In contrast, the BNT162b2 vaccine utilizes 30 micrograms for both priming and boosting. Hence, the potential risk of myocarditis subsequent to mRNA vaccines might be influenced by the mRNA dosage, potentially attributable to the presence of double-stranded RNA capable of inducing dose-related innate immune activation and found in low quantities in mRNA vaccines [68]. The rapid onset of symptoms postvaccination, even after the first dose, aligns with the plausibility of such a direct effect [68].

There is currently no statistical evidence as to whether a lower mRNA COVID-19 vaccine dose will reduce the risk of myocarditis. It is therefore evident that additional research is needed to assess the myocarditis risk related to the COVID-19 lower dose, considering an extended observation period.

### 3.3. Gender-Specific Risk of Myocarditis Associated with the COVID-19 Vaccination

COVID-19 vaccine-induced myocarditis exhibits a male predominance, with a male-to-female ratio of 1.7 : 1, although the underlying cause remains unknown [69, 70]. One possible explanation is related to sex hormones and their interaction with receptors in host cardiac and immune cells [71].

In particular, oestradiol provides a cardioprotective effect by triggering both IL-4-associated Th2-type and anti-inflammatory M2 macrophage responses while promoting mitochondrial fusion in the cardiomyocytes [72]. In contrast, testosterone promotes mitochondrial fission, resulting in an increased production of reactive oxygen species (ROS) and the release of mitochondrial damage-associated molecular patterns (DAMPs) [73]. The outcome involves the activation of the nucleotide-binding and oligomerization domain (NOD), leucine-rich repeat (LRR), and pyrin domain-containing protein 3 (NLRP3) inflammasome. This leads to a pro-inflammatory immune response characterized by the release of IL-1*β* and IL-18, as well as the activation of both M1 macrophages and the Th1 subset of CD4+ T cells [73, 74].

Studies in mouse models of coxsackievirus infection have shown that testosterone promotes TLR4 signalling in myocarditis, which might contribute to increased levels of IL-1*β* and IL-18, leading to cardiomyocyte apoptosis and contractile dysfunction [75, 76]. Indeed, IL-1*β* has demonstrated its role in determining cardiomyocyte apoptosis through the activation of caspase-dependent pathways (via the release of cytochrome C, subsequently activating caspase 3) and caspase-independent pathways (via the upregulation of endonuclease G). Additionally, IL-1*β* inhibits survivin, a member of the inhibitor of apoptosis protein family [77]. Simultaneously, IL-18, through its binding to Toll-like receptors (TLRs), instigates the MyD88 signalling axis. This, in turn, leads to the activation of nuclear factor kappa-light-chain-enhancer of activated B cells (NF-*κ*B) and an augmented production of inducible nitric oxide synthase (iNOS), resulting in diminished myocardial contractile function [78]. Hence, the observed sex differences in the modulation of mitochondrial dynamics and inflammatory responses may contribute to the pathogenesis of COVID-19 vaccine-induced myocarditis, elucidating the higher incidence of this complication in males.

The gender bias is also linked to differences in inflammatory and cardiac biomarkers between genders. A retrospective cohort study by Cheng et al. revealed an independent risk relationship between myocardial injury and inflammatory response in male patients, who are more susceptible to inflammatory stress [79]. The role of angiotensin-converting enzyme 2 (ACE2) in this context is important. ACE2's binding with viral spike proteins not only facilitates SARS-CoV-2 entry into cells but also leads to ACE2 downregulation and uncontrolled activation of the renin-angiotensin-aldosterone system [80]. Notably, the ACE2 gene's location on the X chromosome may contribute to the increased risk of myocardial injury in males due to differences in methylation and sex chromosome activation [81].

In contrast, females tend to have higher oestrogen levels than males, which enhances ACE2 activity and expression in a concentration-dependent manner [82]. This leads to upregulated angiotensin-(1–7) expression, encouraging vasodilation, nitrogen oxide release, and reduced smooth muscle cell proliferation [82, 83]. Oestrogen also plays a protective role against inflammatory injury to the vascular endothelium [84].

### 3.4. Diagnosis

#### 3.4.1. Clinical Presentation

Clinical presentation varies among patients, with some experiencing mild, nonspecific symptoms such as dyspnoea, fatigue, chest pain, and exertional tightness, while others are asymptomatic, possibly due to limited pericardial involvement [43, 85, 86].

In contrast to viral-induced myocarditis, most patients with vaccine-associated myocarditis present with chest pain, but this might reflect a selection bias favoring the identification of symptomatic individuals [54, 87]. Cardiac troponin levels are typically elevated in most patients and peak between 48 and 72 hours after symptom onset [57, 88, 89]. Inflammatory markers, such as C-reactive protein, also show an increase. According to Haussner et al., electrocardiogram (ECG) changes in vaccine-associated myocarditis are subtle, nonspecific, and similar to those in classical myocarditis [90]. These may include nonspecific or mild diffuse ST-segment changes and PR-segment depressions. Some patients may present with sinus tachycardia, while ventricular and supraventricular arrhythmias occur rarely and typically in severe cases [90].

Overall, patients with COVID-19 vaccine-induced myocarditis usually develop a mild disease with favorable outcomes [91]. However, like in classic myocarditis, clinicians must remain vigilant for severe cases, as a limited number of cases have reported associations with fulminant myocarditis characterized by giant cells, eosinophilic myocarditis, and lymphocytic myocarditis [92]. These patients require early detection and vigorous intervention, as discussed in the management section.

**Figure 5:**
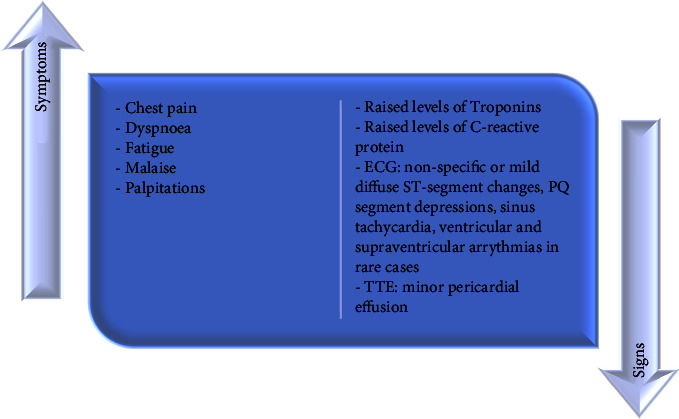
Illustration of the clinical characteristics of COVID-19 vaccine-induced myocarditis. ECG: electrocardiogram; TTE: transthoracic echocardiogram.

Current research suggests that adverse outcomes are rare in patients with vaccine-induced myocarditis [93]. However, acute arrhythmias should be monitored for several days following patient admission. Discharge may occur if there is clinical improvement with no major arrhythmias or deterioration in cardiac function.  Figure 5 illustrates the clinical characteristics of COVID-19 vaccine-induced myocarditis [85].

#### 3.4.2. Endomyocardial Biopsy and Cardiac Magnetic Resonance Imaging

Diagnosing myocarditis can be challenging, with endomyocardial biopsy (EMB) traditionally considered the gold standard [94]. However, EMB is rarely performed in COVID-19 vaccine-associated myocarditis, as cases are often mild [95].

**Figure 6:**
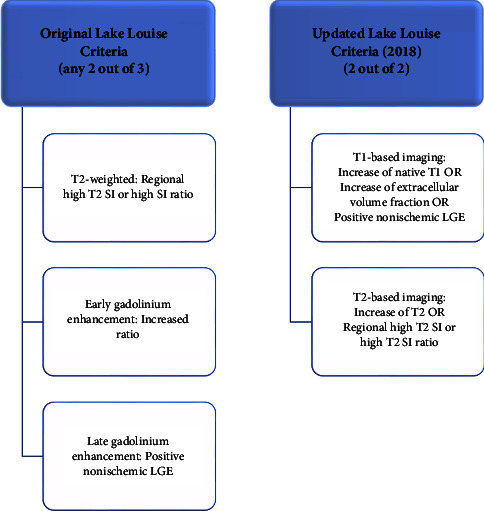
Illustration of the original and updated Lake Louise Criteria that can be used to establish the diagnosis of clinically suspected myocarditis.

Cardiac magnetic resonance (CMR) offers a noninvasive and accurate method for diagnosing clinically suspected myocarditis. The European Society of Cardiology suggests that clinically suspected myocarditis can be diagnosed when clinical symptoms are present, along with at least one of four clinical criteria, including evidence of late gadolinium enhancement (LGE) on CMR [96]. Asymptomatic cases require at least two clinical criteria. Although CMR is highly accurate for diagnosing “infarct-like” myocarditis characterized by fever, chest pain, and elevated ST segments on the ECG, its accuracy is limited in primarily arrhythmic presentations [23]. The updated “Lake Louise Criteria” from 2018 by the International Consensus Group incorporates oedema, T1-mapping, LGE, extracellular volume, and T2-mapping techniques on CMR for myocarditis diagnosis [97].  Figure 6 illustrates the updated Lake Louise Criteria [23].

Cooper et al. emphasize the importance of standard endomyocardial biopsy (EMB) approaches, particularly the left ventricular approach, in diagnosing myocarditis [98]. Additionally, the morphomolecular characteristics of inflammatory myocardial lesions can provide insights into pathophysiology and the clinical course [99].

**Figure 7:**
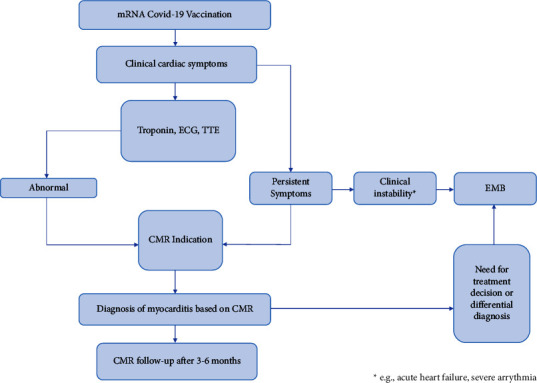
Illustration of the potential workflow of CMR use in patients with suspected COVID-19 vaccine-induced myocarditis. ECG: electrocardiogram; TTE: transthoracic echocardiogram.

Regarding the diagnosis of COVID-19 vaccine-induced myocarditis, reports are limited due to the small number of clinical findings and different referral patterns, often lacking confirmation through CMR or EMB. The accurate number of asymptomatic myocarditis cases following COVID-19 vaccination based on CMR criteria remains unknown, as recent observational studies have not consistently included CMR [100]. Experts recommend that elevated laboratory values, such as troponin, and echocardiogram abnormalities should trigger CMR and, if indicated, EMB interventions [101, 102].  Figure 7 outlines a potential workflow for CMR use in patients with suspected COVID-19 vaccine-induced myocarditis [85].

### 3.5. Pathology of COVID-19 Vaccine-Induced Myocarditis

Due to the low severity of COVID-19 vaccine-induced myocarditis, autopsy reports including histopathological analysis have been limited in number [103, 104]. The clinical community has documented that the majority of cases reflect a lymphocyte predominance, with some cases encompassing additional neutrophil cells [105].

### 3.6. Possible Mechanisms of COVID-19 Vaccine-Induced Myocarditis

#### 3.6.1. Molecular Mimicry of Spike Proteins

One crucial immune mechanism involves molecular mimicry between SARS-CoV-2 spike proteins and self-antigens [105]. Moderna and Pfizer-BioNTech's mRNA vaccines employ lipid nanoparticles for in vitro transcribed (IVT) mRNA delivery encoding the viral spike protein and activating an adaptive immune response [106]. This leads to IgG antibody generation by B-lymphocytes targeting spike proteins, aiding viral neutralization by preventing attachment to ACE2 host cell surface proteins [107].

Some argue that heart-reactive antibodies do not cause myocarditis, but past research suggests they can affect cardiac cells, especially in genetically susceptible individuals [104, 108]. The cross-reactivity of antibodies against viral spike proteins with structurally similar human peptides, including alpha-myosin, raises concerns about vaccine-induced autoimmunity [109]. Research is needed to assess SARS-CoV-2 antigens' potential to induce autoimmunity.

Consider autoantibody generation as a potential mechanism in postvaccination myocarditis for susceptible individuals. Case reports show a peak in IgM and IgG antibodies on day 2 alongside symptoms, without the expected decline as the clinical condition improves [110]. Some argue these autoantibodies may not be pathogenic but a result of myocardial inflammation. Additionally, in the same case reported by Muthukumar et al., there was a twofold increase in natural killer (NK) cell frequency, which remains unclear in its contribution to disease resolution or pathology [110].

#### 3.6.2. Overactive Immune System Response

Research on mRNA vaccines highlights their potential to induce myocarditis through excessive immune responses [106]. Prior studies proposed that RNA components and lipid nanoparticles in COVID-19 vaccines can trigger an exaggerated innate immune response, potentially causing vaccine-associated myocarditis [111]. Despite being the first clinical use of IVT mRNA, mRNA vaccines faced early challenges due to mRNA molecule instability and inherent immunogenicity. Wolff et al. investigated endosomal Toll-like receptors in immune cells and cytosolic receptors such as TLR3, TLR7, TLR8, RIG-I, and MDA5's potential to cross-react with IVT mRNA [112]. Activation of these receptors initiates an inflammatory cascade, leading to inflammasome assembly, type I interferon production, and NF-*κ*B translocation [113].

Recent literature suggests lipid nanoparticles may trigger TLR-mediated pro-inflammatory cytokine release and complement activation-related hypersensitivity reactions [113]. These findings show that overly aggressive immune responses could contribute to COVID-19 vaccine-induced myocarditis.

### 3.7. Management of Vaccine-Related Myocarditis

Regarding management, most cases of COVID-19 vaccine-induced myocarditis are self-limiting [114]. However, clinicians must assess myocardial risk, especially in young males with postvaccination chest pain. Initial evaluation includes interpreting ECG, cardiac troponin, and inflammatory marker levels [115]. Individuals with suspected COVID-19 vaccine-induced myocarditis should consider consultation with a cardiologist and assessment using CMR and TTE [115]. Managing arrhythmias and heart failure associated with the COVID-19 vaccination should also be addressed [97].

For arrhythmias and heart failure, guideline-directed therapies recommend heart failure drugs such as angiotensin-converting enzyme inhibitors (ACE-Is), angiotensin receptor blockers (ARBs), beta-blockers, sodium-glucose cotransporter 2 inhibitors, and mineralocorticoid receptor antagonists [116].

There is controversy regarding ACE-Is and ARBs due to early reports from China highlighting that patients with hypertension exhibited worse outcomes [117, 118]. However, analyses were crude and cofounders, i.e., older age and cardiovascular disease, associated with hypertension were also present [119]. ACE-Is could theoretically increase SARS-CoV-2 infection risk due to ACE2's role as a binding site [120]. Although these drugs do not directly affect ACE2 activity, animal studies suggest they may upregulate ACE2 in the heart, raising concerns about COVID-19 susceptibility [121]. However, no human study supports this hypothesis [120]. In turn, the European Society of Cardiology issued the position statement that ACE-Is/ARBs should not be discontinued, while current evidence suggests that there is no significant association between these agents with COVID-19 diagnosis or worse outcomes [122, 123].

In fulminant myocarditis cases, characterized by severe hemodynamic instability, the American Heart Association recommends EMB as a class 1 indication [124, 125]. Mechanical circulatory support can also benefit cases with left ventricular dysfunction.  Table 5 summarizes the management of COVID-19 vaccine-induced myocarditis.

Some published case reports have used corticosteroids and colchicine to manage patients, including those with persistent mild symptoms. Hajjo et al. proposed an approach that carefully weighs the benefits and risks of immunosuppression in COVID-19 vaccine-induced myocarditis, suggesting the selective use of corticosteroids, particularly glucocorticoids, for a limited duration in patients with acutely impaired left ventricular function [126–128]. Additionally, aspirin, intravenous immunoglobulin, beta-blockers, and angiotensin-converting enzyme inhibitors may be considered for patients with left ventricular systolic dysfunction [129, 130].

### 3.8. Recovery and Surveillance

Regarding the recovery from COVID-19 vaccine-induced myocarditis, several dilemmas exist [129]. A study from the Multicenter Lombardy Registry in 2018, which examined 429 adult patients who survived acute myocarditis, found that only 4.5% of them had residual left ventricular dysfunction at the 3-year follow-up [130]. As myocarditis can have long-term consequences, postacute care involving laboratory tests, ECG, and TTE is essential [130]. Furthermore, there is limited evidence available on the effects of exercise restriction for 3–6 months postvaccine-induced myocarditis on recovery and the prevention of sudden cardiac death [131].

## 4. Discussion

Extensive research has established a clear association between the mRNA COVID-19 vaccination and myocarditis. Diaz et al. [53] reported a distinct myocarditis syndrome primarily occurring after the second vaccine dose, with an incidence of 1.0 per 100,000 individuals. Albert et al. [63] also supported this link, particularly among males aged 16 to 30. The literature review explained the male predominance by examining gender-based differences in inflammatory pathways, cardiac biomarkers, and sex hormones.

Additionally, the lack of a definite immunopathophysiological mechanism underscores the need for further exploration to understand the reasons behind COVID-19 vaccine-induced myocarditis, differentiating it from infection-related myocarditis and assessing the impact of elevated cytokine levels on various organs, as observed in COVID-19 infection.

Furthermore, a detailed analysis of diagnostic and management strategies highlights the absence of guidelines for acute patient presentations. Challenges include determining the appropriate timeframe for guideline-driven therapy and managing cases with persistent symptoms but without significant cardiac abnormalities or troponin elevations. Questions also arise about administering the second vaccine dose to individuals with COVID-19 vaccine-induced myocarditis after the first dose and selecting the appropriate vaccine agent.

### 4.1. Limitations

Firstly, due to the emergence of COVID-19 vaccine-induced myocarditis in early 2021, there is a lack of prior research on this topic. Consequently, epidemiological data are limited, primarily sourced from a few countries such as the USA, France, Denmark, the UK, and Israel. Furthermore, studies reporting these data may exhibit selection bias, often relying on cohort and case-controlled studies for participant selection, overestimating the cases of myocarditis.

The proposed hypotheses may lack strong direct empirical evidence and often rely on limited data from case series. Additionally, the mild presentation of affected individuals has contributed to the scarcity of invasive investigations, such as endomyocardial biopsy (EMB).

### 4.2. Recommendations for Research

Based on the analysis and literature review of COVID-19 vaccine-associated myocarditis, the following recommendations are proposed:

1. Immunopathological Mechanisms Research: future investigations should aim to confirm the immunopathophysiological mechanisms of COVID-19 vaccine-induced myocarditis. This research should determine whether these mechanisms are unique to mRNA vaccination or associated with spike protein delivery via mRNA. Strategies for reducing inflammatory vaccine reactions could involve modifying IVT mRNA components, utilizing modified nucleosides to mitigate innate immune responses, and eliminating dsRNA by-products and abortive RNA transcripts. Although some experimental therapies have explored innate immune inhibitors with IVT mRNA, these approaches have not yet been applied to the two distinct mRNA COVID-19 vaccines. Redesigning lipid nanoparticles may also assist in reducing immunogenicity. It is essential to strike a balance between these efforts and the necessity of generating a robust immune response for effective vaccine protection.
2. Role of Immune Cell Populations: research should prioritize elucidating the roles played by specific immune cell populations in post-COVID-19 vaccine immunization, infection, myocardial injury, and COVID-19 vaccine-associated myocarditis. A comprehensive understanding of how different immune cells contribute to these processes can provide valuable insights into vaccine safety and efficacy.
3. Risk-Benefit Analysis for Different Demographics: subsequent studies should thoroughly investigate the potential risks and benefits of COVID-19 vaccination across various gender and age groups, with a specific focus on different vaccine doses. Prospective screening for myocarditis following the mRNA COVID-19 vaccination should target diverse population groups, paying particular attention to gender and age-related characteristics, notably among young males. Additionally, highly physically active individuals may benefit from cardiac screening, even in the absence of symptoms, to detect significant cardiac complications.

## 5. Conclusion

In summary, this literature review aimed to explore the link between myocarditis and mRNA COVID-19 vaccination. The research reviewed indicates that myocarditis is indeed a rare outcome of the mRNA COVID-19 vaccination. While the precise mechanistic explanations remain uncertain, the roles of spike proteins and inflammatory cytokines should not be disregarded pending further investigation. Additionally, the observed male predominance in COVID-19 vaccine-related myocarditis may have implications for assessing the risk-benefit ratio of subsequent mRNA COVID-19 vaccine doses. Future research efforts should prioritize investigating this association using a collaborative registry approach that collects comprehensive data on patient demographics, clinical presentation, laboratory biomarkers, imaging findings, and other relevant investigations. This will provide clearer guidance on managing myocardial injury associated with the mRNA COVID-19 vaccination.

## Figures and Tables

**Table 1:** Overview of the inclusion and exclusion criteria used in the database search.

Inclusion criteria	Exclusion criteria
Primary research	Scholarly journals
Primary research: qualitative, quantitative, mixed-methods studies	Literature not available in English
Peer-reviewed papers	Date of publication: prior to January 1, 2000
Language: English written literature	Considers any other types of COVID-19 vaccination
Date of publication: January 1, 2000, to December 31, 2023	Considers other adverse effects post-COVID-19 vaccination
Considers mRNA type of COVID-19 vaccination only	
Considers myocarditis post-COVID-19 vaccination	

**Table 2:** Overview of the baseline characteristics of the studies.

Reference	Publication year	Country	Type of study	Mean age (years)	Sample size	Follow-up (days)	Male (% total)	mRNA vaccine type	Number of myocarditis cases	Male (% myocarditis)	Myocarditis diagnostics	Number of hospitalizations	Number of deaths
El Sahly et al. [49]	2021	USA	Population study	51	14287	14	53	Moderna	0	0	N/A	0	0
Walter et al. [50]	2022		Population study	8	1518	7	52	Pfizer	0	0	N/A	0	0
Simone et al. [51]	2021		Population study	49	2392924	10	46	Pfizer/Moderna	15	100	Clinical	0	0
Ali et al. [52]	2021		Population study	14	2489	83	52	Moderna	1	0	N/A	0	0
Diaz et al. [53]	2022		Population study	57	2000287	N/A	41	Pfizer/Moderna	20	75	Abnormal troponin or CMR evidence	19	0
Montgomery et al. [54]	2021		Population study	25	2810000	N/A	100	Pfizer/Moderna	23	100	Clinical	0	0

Le Vu et al. [55]	2022	France	Population study	28	18129	30	61	Pfizer/Moderna	82	79	Hospital admission codes	N/A	N/A

Husby et al. [56]	2022	Denmark	Population study	N/A	3482295	28	N/A	Pfizer	48	73	Clinical diagnosis + troponin elevation + hospitalization >24 hours	28	1
			Population study	N/A	498814	28	N/A	Moderna	21	N/A	Clinical diagnosis + troponin elevation + hospitalization >24 hours	8	0

Patone et al. [57]	2022	UK	Population study	56	16993389	28	33	Pfizer	158	67	Hospital admission codes	N/A	N/A
			Population study	40	1006191	28	26	Moderna	9	83	Hospital admission codes	N/A	N/A

Witberg et al. [58]	2021	Israel	Population study	44	2500000	42	49	Pfizer	54	94	Clinical	1	1
Mevorach et al. [59]	2021		Population study	33	5000000	30	49	Pfizer	136	91	Clinical	114	1
Barda et al. [46]	2021		Population study	38	884828	42	52	Pfizer	21	91	Clinical	N/A	N/A

**Table 3:** Report of the observed number of myocarditis cases in a 7-day risk window after the second dose of mRNA COVID-19 vaccination according to USA VAERS through June 11, 2021.

Age group (years)	Average doses administered	Observed cases	
		Females	Males
12–17	2114799	19	128
18–24	4787275	23	219
25–29	3888775	7	59
30–39	8833799	11	61
40–49	9252770	18	34
50–64	17476189	18	18
>65	19875261	10	11

**Table 4:** Report of myocarditis crude rates and related deaths per million mRNA vaccine doses administered by age, sex, and dose number to VAERS following mRNA COVID-19 vaccination through June 11, 2021.

Age group (years)	Female rates per million doses			Male rates per million doses			Death reports per million doses administered
	All doses	Dose 1	Dose 2	All doses	Dose 1	Dose 2	
12–17	4.2	1.1	9.1	32.4	9.8	66.7	1.1
18–24	3.6	1.5	5.5	30.7	8.7	56.3	1.3
25–29	2.0	0.8	2.6	12.2	4.5	20.4	1.3
30–39	1.8	1.4	1.8	6.9	2.0	10.0	2.4
40–49	2.0	0.9	2.8	3.5	1.0	5.1	3.8
50–64	1.6	1.0	1.8	1.9	1.0	2.3	6.9
>65	1.1	0.6	1.2	1.2	0.7	1.4	14.4

**Table 5:** Summary of management of COVID-19 vaccine-induced myocarditis.

Clinical presentation	Treatment
Chest pain	Initial evaluation using ECG, cardiac troponin, and inflammatory marker levels [114]

Arrhythmias	Guideline-directed therapy based on arrhythmia type [115]

Heart failure with reduced ejection fraction	Angiotensin-converting enzyme inhibitors
	Angiotensin receptor blockers
	Beta-blockers
	Sodium-glucose cotransporter 2 inhibitors
	Mineralocorticoid receptor antagonists [115]

Fulminant myocarditis-cardiogenic shock	Short-term corticosteroids
	Mechanical circulatory support in left ventricular dysfunction [123, 124]

## Data Availability

The primary databases employed in the sourcing of material in this review were PubMed, Embase, and Queen Mary University of London Library Services.

## Acronyms

ACE-Is: Angiotensin-converting enzyme inhibitors
ACE2: Angiotensin-converting enzyme 2
APCs: Antigen-presenting cells
ARB: Angiotensin receptor blocker
BNT162b2: Pfizer-BioNTech vaccine
CDC: US Centers for Disease Control and Prevention
CMR: Cardiac magnetic resonance
COVID-19: Coronavirus disease 2019
DAMPs: Damage-associated molecular patterns
EAM: Electroanatomic mapping
ECG: Electrocardiogram
EMB: Endomyocardial biopsy
FDA: US Food and Drug Administration
IFN-*γ*: Interferon gamma
IL: Interleukin
iNOS: Inducible nitric oxide synthase
IVT: In vitro transcription
LGE: Late gadolinium enhancement
LRR: Leucine-rich repeat
MDA 5: Melanoma differentiation-associated protein 5
MHC: Major histocompatibility complex
mRNA-1273: Moderna vaccine
mRNA: Messenger RNA
NF-*κ*B: Nuclear factor kappa-light-chain-enhancer of activated B cells
NK: Natural killer
NLRP3: Pyrin domain-containing protein 3
NOD: Oligomerization domain
RIG-I: Retinoic acid-inducible gene I
ROS: Reactive oxygen species
SARS-CoV-2: Severe acute respiratory syndrome coronavirus-2
Th: T-helper cell
TLR4: Toll-like receptor 4
TNF-*α*: Tumour necrosis factor-alpha
TTE: Transthoracic echocardiography
VAERS: Vaccine Adverse Event Reporting System
WHO: World Health Organization.
